# Tereza de Jesus Sena: reformer of psychiatric and mental health nursing education and practice

**DOI:** 10.1590/0034-7167-2025-0223

**Published:** 2026-07-24

**Authors:** Maria Angélica de Almeida Peres, José Lúcio Costa Ramos, Priscilla Ingrid Gomes Miranda, Lygia Paim, Cristina Maria Douat Loyola

**Affiliations:** I Universidade Federal do Rio de Janeiro. Rio de Janeiro, Rio de Janeiro, Brazil; II Universidade Federal da Bahia. Salvador, Bahia, Brazil; III Universidade Ceuma. São Luís, Maranhão, Brazil

**Keywords:** Nursing, Biography, History of Nursing, Psychiatric Nursing, Mental Health., Enfermería, Biografía, Historia de la Enfermería, Enfermería Psiquiátrica, Salud Mental.

## Abstract

**Objectives:**

to describe the biography of Tereza de Jesus Sena, highlighting her contributions to psychiatric and mental health nursing education and practice.

**Methods:**

this biographical study used historical analysis to triangulate sources and interpret the chronologically organized data in context. Data were collected from August to December 2023.

**Results:**

three categories emerged: Personal data, education, and early professional career; Formal appointment and recognition as a Psychiatric Nursing instructor; and Sena’s relationship with the development of psychiatric nursing in Brazil.

**Final Considerations:**

the Ceará-born nurse stood out for her work in the states of Bahia, São Paulo, and Rio de Janeiro. She devoted herself to her work and to disseminating knowledge through education as tools to transform the psychiatric and mental health field, becoming a mentor of subjectivity-attuned care and of modern teaching for her time.

## RESUMEN

**Objetivos:**

describir la biografía de Tereza de Jesus Sena, destacando su contribución a la docencia y la práctica en enfermería psiquiátrica y salud mental.

**Métodos:**

estudio biográfico; el análisis histórico trianguló fuentes e interpretó los datos de forma contextual, organizados cronológicamente. La recogida se realizó entre agosto y diciembre de 2023.

**Resultados:**

se identificaron tres categorías: Datos personales, formación e inicio de la trayectoria profesional; Nombramiento y reconocimiento como docente de la asignatura de Enfermería Psiquiátrica; y Relación de Tereza Sena con el desarrollo de la enfermería psiquiátrica en Brasil.

**Consideraciones Finales:**

la enfermera nacida en Ceará se destacó por su labor en Bahía, São Paulo y Río de Janeiro. Apostó por el trabajo y la difusión del conocimiento mediante la educación como herramientas transformadoras del ámbito psiquiátrico y de la salud mental, convirtiéndose en referente de un cuidado centrado en la subjetividad y de una docencia innovadora en su tiempo.

## INTRODUCTION

Mental health is currently an interdisciplinary field, with teams working in defined geographic areas and responsible for providing care focused on the well-being of people experiencing psychological distress^(1)^. Understanding the advances that have driven the consolidation of mental health nursing in the 21st century calls for studies that contextualize the development of knowledge and practices. Such work helps explain how Brazilian nursing assumed a leading role in a model of care that shifted from psychiatric institutions to community-based services.

Records of the Anti-Asylum Movement in Brazil date back to the 1960s, when society-represented by people who use mental health services, family members, and mental health workers-mobilized against an inhumane and incoherent reality of psychiatric care. Asylum-based practices were denounced across various media outlets, echoing a call for transformation that remains ongoing^(2)^.

It was in the 1960s that a nurse committed to the care of people experiencing psychological distress gained national visibility for her courage, intelligence, and competence, placing her among the leading professionals of her time. Tereza de Jesus Sena (spelled Teresa in some sources) became an example for generations who came to know her work as a nurse, educator, researcher, and manager; her biography can undoubtedly illuminate and inspire future generations of professionals in the field of mental health care^(3)^.

The rationale for this biographical study is to highlight Tereza de Jesus Sena’s contributions to Brazilian nursing, especially in psychiatric and mental health nursing. The intent is to preserve her memory and make her life and work known to society; her dedication to specialized care unfolded at a time when transformations in mental health practices were still constrained by an asylum-based culture that, through psychiatric institutions, excluded people experiencing psychological distress from society^(2)^.

Her work remains a foundation for supporting dignified care for people hospitalized in psychiatric hospitals and for fostering awareness among future professionals through psychiatric nursing education; in doing so, it reflects a reality gradually reshaped by collective and individual struggles to secure recognition for the specialty and reduce the stigma directed at professionals in this field^(4)^. She was a woman recognized for the excellence of her work, advancing clinical care through specialized knowledge, scientific research, and an ability to understand others-whether the person experiencing psychological distress, their family, or other health professionals.

## OBJECTIVES

To describe the biography of Tereza de Jesus Sena, highlighting her contributions to psychiatric and mental health nursing education and practice.

## METHODS

### Ethical considerations

Because this was historical research based on publicly accessible documentary sources, review by a Research Ethics Committee was not required.

### Study design

A qualitative biographical study^(5)^. In this type of study, the typical characteristics of an individual whose trajectory has contributed to collective knowledge and the advancement of science are described in detail^(6)^. We followed the Standards for Reporting Qualitative Research (SRQR)^(7)^.

### Data collection and organization

The documentary corpus consisted of school records, reports, registry books, photographs, and graduation invitations consulted at the Museum of the School of Nursing at the Federal University of Bahia. We also included speeches, articles, news reports, official correspondence, and two interviews with Sena (one in 1990 and another in 2002) from the holdings of the Documentation Center at the Anna Nery School of Nursing, Federal University of Rio de Janeiro. Data were collected from August to December 2023.

### Data analysis

The analysis involved source triangulation, conducted after document selection, chronological organization, and categorization. Data were grouped into themes: biographical data, educational background, and professional work. The final stage involved historical description and interpretation, culminating in a synthesis of the text into three categories: Personal data, education, and early professional career; Formal appointment and recognition as a Psychiatric Nursing instructor; and Sena’s relationship with the development of psychiatric nursing in Brazil.

## RESULTS

### Personal data, education, and early professional career

Tereza de Jesus Sena, the daughter of Francisco Sena Araújo and Júlia Adélia Araújo, was born on October 8, 1923, in Acaraú, in the state of Ceará-238 km from Fortaleza, the state capital. In Fortaleza, she completed her primary education at Colégio Estadual do Ceará and her secondary education at Escola Normal Justiniano de Serpa, traditional and highly regarded institutions in the state^(8)^.

Her first higher education degree was in Pharmacy, from the School of Pharmacy and Dentistry of Ceará. She earned her nursing degree at the Bahia School of Nursing, where she lived in the school residence hall. Her school records indicate that she entered the program on March 15, 1949, and graduated on December 9, 1952. By then, both of her parents had died.

During her time off, according to entries in the residence hall’s Students’ Entry and Exit Logbook (1950-1951), Tereza Sena would go to the post office, downtown, the cinema, and Farol da Barra; these outings suggest that she kept in touch with family and friends by letter and that she sought out cultural activities.

Her graduation, along with that of 25 classmates, took place during a commencement ceremony and religious services on December 9 and 10, 1952. After graduating, she completed a 1 month training program at the Psychiatric Clinic of the University of São Paulo (USP). On returning to Bahia, she was hired as a faculty member at the School of Nursing of the Federal University of Bahia (UFBA), where she taught Psychiatric Nursing, Neurology, and Neurologic Nursing between 1953 and 1955. She also supervised students during their Psychiatric Nursing clinical placement at Juliano Moreira Hospital, founded in 1869 in the city of Salvador^(9)^.

From 1954 to 1955, Tereza Sena served as Head of the Nursing Service (Nursing Division) at UFBA’s Professor Edgard Santos University Hospital, also known locally as Hospital das Clínicas. She worked with Nelson Soares Pires, chair of psychiatry at the Bahia School of Medicine and recognized for initiating psychiatric reform in Bahia, to organize the hospital’s Psychiatric Unit. Inaugurated in 1953, this unit was the first psychiatry service in a general hospital in Brazil, consisting of an all-female ward that admitted patients from all social classes. With her innovative approach, Tereza Sena became responsible for the nursing service on this psychiatric ward, and physicians also provided on-site outpatient consultations^(10)^.

In 1956, she was hired by the Department of Assistance to Psychopaths (*Departamento de Assistência ao Psicopata*, DAP; historical name) in São Paulo as director of nursing at *Hospital Central do Juqueri* (Juqueri Central Hospital), an institution created in the 19th century that at the time operated as a psychiatric hospital-colony with 5,210 beds. Juqueri was a national reference between 1923 and 1938 under the direction of Antônio Carlos Pacheco e Silva. Its facilities were progressively expanded, and the staff included general practitioners, neurologists, and psychiatrists^(11)^. When Tereza Sena arrived to work as a nurse, Juqueri was operating at more than twice its capacity: more than 11,000 institutionalized patients were distributed across 12 pavilions with male, female, and pediatric wards, an asylum wing, and a medical center. The institution also housed a forensic psychiatric unit, a cemetery, and other facilities^(12)^.

During her work at the Juqueri Hospital-Colony, she met the American nurse Verna Fraser, who had been sent to Brazil by the Special Public Health Service (SESP) to serve for 2 years as a psychiatric nursing consultant in nursing schools, since the psychiatry rotation had become mandatory. The American consultant recommended Tereza for a master’s degree in Psychiatric Nursing, supported by a United States government scholarship. Tereza Sena recounted her first meeting and her subsequent work with this nurse:

*I had been there [at Juqueri] only a short time when an American psychiatric nursing consultant arrived: Verna Fraser was her name; she did not speak a word of Portuguese, and I did not speak English, nor did I understand it, and I began to learn with her [...]. And we would also work until late at night because she would go there for periods in São Paulo [...]. And one day, out of the blue, she asked me: Tereza, if I can get you a scholarship from the American government to go to the United States, would you like to go take a course? If you accept, you will be my nominee* ^(13)^.

After 10 months devoted to learning English, Tereza Sena was interviewed by United States government representatives and, in January 1958, traveled to Detroit, Michigan. She was the only psychiatric nurse nominated for the scholarship during that period. She completed a master’s degree in Psychiatric Nursing at Wayne State University College of Nursing, along with clinical placements at St. Elizabeths Hospital in Washington and at the Health, Education, and Comfort Department in New York. She returned to Brazil in 1959, becoming director of nursing at Juqueri Hospital and a clinical placement supervisor for students from the USP School of Nursing, consolidating her knowledge and career in psychiatric and mental health nursing.

### Formal appointment and recognition as a Psychiatric Nursing instructor

Tereza Sena’s teaching career began in Bahia soon after her graduation, alongside her clinical work. When she moved to São Paulo, she began supervising students from the USP School of Nursing during their clinical placement at the Juqueri Hospital-Colony, maintaining the link between teaching and clinical care.

However, Tereza hoped to move to Rio de Janeiro and join the Anna Nery School of Nursing at the University of Brazil (UB), a national reference at the time. That opportunity arose unexpectedly, after the school’s director, Waleska Paixão, called the Juqueri Hospital-Colony to speak with the director of nursing. Tereza Sena recalled that the conversation was direct:

*Dona Waleska sent for me, and I went to Rio on vacation; she talked to me and said she needed a professor [...]. But I told her that I wanted to hand over all my work first. I had already secured six nurses there [at Juqueri], but Dona Waleska said she couldn’t wait, so I left a nurse in my place* ^(13)^.

Her move to the Anna Nery School was hindered by the refusal of the DAP-SP to release her for transfer. On December 31, 1959, after negotiations led by Director Waleska Paixão, Tereza was hired by the National Campaign Against Tuberculosis (CNCT), which could admit staff temporarily for 1 year. The arrangement between the Anna Nery School and the CNCT involved Tereza’s secondment in exchange for the nurse Ariadne Lopes de Menezes, whom the School had long seconded to the Campaign.

In April 1960, Tereza Sena began teaching the Psychiatric Nursing course at the Anna Nery School. Her integration was not easy because she was from Brazil’s Northeast and had trained at another school, as she reported in her interview when recalling how the Anna Nery faculty received her:

*To be honest, [I was received] very badly-indifferently; socially, they didn’t want to talk to me, and they even criticized me. Sometimes I found out through the students, but I always ignored it; I never even mentioned it to Dona Waleska [...] there was a covert boycott* ^(14)^.

Despite her colleagues’ rejection, she continued to devote herself to psychiatric nursing education, while the school director worked to secure her permanent appointment through negotiations with health service directors and the UB rector. In February 1961, she was appointed course coordinator under UB’s extraordinary staff. She was initially hired for 3 months and paid through a special allocation. This arrangement was maintained through contract renewals every 4 months until 1962, when she was formally appointed as a nurse under UB’s extraordinary staff^(3)^.

Her appointment to the university’s regular staff came in 1969, under Law No. 3,780 of July 12, 1960, as amended by Law No. 4,069 of June 11, 1962, which established that civil servants with 5 years of continuous service in permanent activities would be placed into the university’s positions and roles^(15)^.

Tereza Sena began covering for physician faculty by teaching the Mental Hygiene and Clinical Foundations of Psychiatry courses (formerly known as the Psychiatry course). When assessing how these courses had been taught up to that point, she described it as “fifty years behind”^(14)^.

She modernized the teaching with new theoretical and practical perspectives on caring for people experiencing mental distress in institutional settings, and she built an affective bond with students-eventually inspiring them to pursue psychiatry. A former student interviewed in another study stated:

*I loved the nursing program [...]. And then... I mean, I already had a tendency, and then someone came along who really helped me decide, and that was Tereza Sena. She gave those wonderful classes, and from the time I spent with Tereza, I was already leaning that way. So, that was my destiny [working as a psychiatric nurse]* ^(13)^.

Professor Tereza Sena also inspired future nurses through her professional presentation. She wore a white uniform and kept her hair neatly styled. Through this, she taught the importance of a well-groomed, harmonious professional presentation-even when, at times, she was dealing with the disorganization that can accompany psychosis. She related to patients with warmth and humor, sometimes laughing with them about their stories. She never laughed at patients, reflecting her professional ethics.

She had training in psychoanalysis^(4)^, so in the 1970s she taught using concepts from Jacques Lacan. She guided students on how to accompany and, in Lacanian terms, “be a secretary to” the psychotic experience of patients with schizophrenia, some of whom were difficult to approach; and she worked from the premise that the patient was the one who knew themselves. The person accompanying the patient should follow them through their delusions or hallucinations, since their behaviors bore witness to their mental health. Every morning, at the end of the clinical placement, there was a meeting with the professor to discuss cases and the approaches students had taken.

In her contact with patients, she applied Joyce Travelbee’s Person-to-Person Relationship Model, intended to help individuals cope with illness and suffering and, when possible, find meaning in these experiences-ultimately fostering hope. Students learned that mental health care was not epic-that is, there was no triumphant arrival at a cure. Through small gains, they came to recognize what was possible for each patient.

In the Anna Nery School of Nursing’s graduate program, launched in 1972 with a master’s program, Tereza Sena was part of the pioneering faculty-alongside Elvira de Felice Souza, Maria Dolores Lins de Andrade, Cilei Chaves Rhodus, and Vilma de Carvalho-responsible for planning and implementing the program. She contributed to stricto sensu graduate education in nursing by supervising research in psychiatry and other fields^(16)^. In 1989, with the establishment of the doctoral program in nursing at EEAN, she began supervising dissertations. From 1983 to 1990, she served as coordinator of EEAN’s stricto sensu graduate program.

Tereza Sena also exerted a major influence on the development of graduate nursing education at the Federal University of Ceará (UFC) during the consolidation phase of stricto sensu graduate programs in Brazil. Her role as a research supervisor and scientific consultant in program studies underscored the institutional dialogue established at the time between training centers in Brazil’s Southeast-especially EEAN/UFRJ-and emerging initiatives in the Northeast. This partnership supported the circulation of theoretical and methodological frameworks, strengthened the quality of the studies produced, and contributed to the development of local faculty.

In addition, she played an active role in scientific events as an organizer, course instructor, discussant in discussion groups, and presenter^(17)^; published a book on psychiatric nursing^(18)^; published articles^(19,20)^; served on the committee that evaluated submissions for Revista Brasileira de Enfermagem^(21)^; and sat on examination committees for public competitive examinations for faculty positions^(22,23)^ and for habilitation (*livre-docência*) examinations^(24)^. She also served on the Ministry of Education and Culture (MEC) Nursing Education Specialists Committee and worked as a consultant to the Department of University Affairs^(25)^. One of her achievements in this role was the feasibility and implementation study for UFC’s nursing program, created in 1970^(26)^.

Teaching and clinical practice in psychiatric and mental health nursing went hand in hand throughout Tereza Sena’s trajectory in Bahia, São Paulo, and Rio de Janeiro. These complementary efforts had positive impacts on health services and on nurses’ education. She received many honors as the class patron for graduating cohorts, writing speeches for these occasions on topics related to psychiatric nursing; these speeches were archived by EEAN as primary sources and merit further historical research.

### Sena’s relationship with the development of psychiatric nursing in Brazil

As part of efforts to improve teaching quality at the Anna Nery School, linking patient care and teaching had been advanced through the appointment of nurses and School faculty to nursing leadership roles in hospitals, as occurred at Hospital São Francisco de Assis, the Teaching Maternity Hospital, and the UB Institute of Neurology. Accordingly, after Tereza Sena secured a permanent appointment at the university, Director Waleska Paixão worked with José Leme Lopes, director of the UB Institute of Psychiatry (IPUB), to have Sena appointed as director of nursing^(14)^. It is worth noting that, up to that point, IPUB had never had a formally trained nurse on staff^(4)^.

From 1962 onward, as IPUB’s director of nursing, Tereza Sena began restructuring psychiatric nursing care at the institution, especially in the inpatient wards; this area still lagged behind the “New Psychiatry” approach already established in other countries (e.g., the United States), where she had completed her master’s training and from which she had brought back a new vision of psychiatric nursing^(27)^.

At the same time, she began reconfiguring IPUB’s care environment in ways that ran counter to the asylum model practiced there:

*For them [the nursing team], what mattered was medicating the patient, restraining the patient, locking them in the seclusion room, and assuming the patient was simply expected to put up with it* ^(13)^.

Tereza Sena described how she negotiated ending the use of the seclusion room:

*One day, I went to the administrator and asked why there was still a seclusion room back then. He said: It’s because there’s no one here who will take responsibility. Then I told him I would take responsibility [...]. So, for several nights, I went to the Institute and worked the night shift so the patients could sleep with the door open* ^(14)^.

Beyond closing the seclusion room, she introduced other care changes, aligning IPUB nursing practice more closely with practices in more advanced countries. To do so, she faced resistance from the nursing and nutrition teams:

*Everything was forbidden; [the kitchen staff] would laugh and think it was all crazy stuff; no one ate from a plate or used a spoon [...]. I also began to show the nutritionist that they could use utensils. [...] they started trying it, and I stayed on top of it* ^(14)^.

New care practices involved recognizing hospitalized patients as individuals, without coercive surveillance, and included outings and other activities such as gardening workshops. Tereza Sena received the students in the morning and, in the afternoon, oversaw the administration of the nursing service^(14)^. She also influenced the hiring of nurses trained at the Anna Nery School for IPUB, helping build a professional identity grounded in a modern outlook on nursing care for people with mental health conditions.

Her actions reverberated across nursing schools in Brazil, which began requesting affiliation with the Anna Nery School so their students could enroll in the Psychiatric Nursing and Art of Nursing courses^(4)^. In this context, the affiliated schools were: the Luiza de Marillac School of Nurses (Pontifical Catholic University of Rio de Janeiro), the Alfredo Pinto School of Nursing (Ministry of Health), and the Wenceslau Brás School of Nursing (Itajubá, Minas Gerais).

In 1963, given the high number of affiliations, it was agreed that school directors would first send faculty members for training and then send students. In this way, the faculty members themselves supervised the clinical placement at IPUB, while the theoretical classes continued to be taught by Tereza Sena^(3)^.

The presence of Anna Nery School students was important for implementing the new nursing model at IPUB because, under Professor Tereza Sena’s guidance, they took part in the change process. With a grounding in psychoanalysis, she was adept at facilitating student-professor and student-patient relationships, as evidenced by her statement:

*They [the students] loved me. I don’t even know whether they liked psychiatric nursing that much, because they still had all that fear of the patient. [...] and the next day I would talk with them so they could tell me what they thought, how the student managed to convey something to the patien* ^(14)^.

Educational work was framed as an expression of subjectivity, with this dimension cultivated through dialogue between professor and student. The aim was for novice students to understand their place in that setting and their role as trainees-an approach that both welcomed them and reflected the professor’s concern for everyone’s well-being. At the time, bringing the subjective dimension into teaching and clinical care was an explicit goal at IPUB, as the medical staff sought to apply psychoanalytic concepts more intensively-concepts already widely used by U.S. psychiatrists-while introducing research in this area and changing the care delivered at the institution^(27)^.

Tereza Sena remained IPUB’s director of nursing and the faculty member responsible for psychiatric nursing education for 18 years. She left the position to join the faculty of Brazil’s first master’s program in nursing, launched in 1972 at the Anna Nery School - already known as Anna Nery Nursing School (EEAN)^(3)^.

Throughout her career, Tereza was a full member of the Brazilian Nursing Association (ABEn) and actively engaged in professional advocacy. In 1977, for example, she coordinated the Theme Committee of the 29th Brazilian Nursing Congress in the state of Santa Catarina. She also served as a councilor on the Federal Nursing Council (Cofen), as part of the executive board elected for the 1985-1988 term, chaired by nurse Ivanete Alves do Nascimento^(28)^.

In 2000, after returning to live in Fortaleza, she was honored by IPUB, which named one of its wards after her, preserving the memory of her contributions to psychiatric and mental health nursing.

Tereza de Jesus Sena died of Alzheimer’s disease on May 24, 2004, at age 81, at Charity Hospital (Hospital de Caridade) in Florianópolis, Santa Catarina. Her body was transported to Fortaleza, Ceará, where she was buried.

In 2023, the Ceará Academy of Nursing (Academia Cearense de Enfermagem, ACEN), founded on February 12, 2011, documented the history of nurses who played important roles in care, teaching, and research in the state through the book Academia Cearense de Enfermagem (2007-2023). The chapter on Tereza de Jesus Sena reports that, on August 22, 2013, Chair No. 12 was created, for which she is the patron. The current occupant is retired professor Maria de Nazaré de Oliveira Fraga of UFC, also a mental health specialist and Sena’s master’s advisee at EEAN from 1977 to 1981^(28)^.

## DISCUSSION

As a leading figure in Brazilian nursing, Tereza Sena rose to national prominence through her work in Bahia, São Paulo, and Rio de Janeiro, where she helped advance psychiatric and mental health nursing knowledge and practice. Her trajectory shows that she worked with equal competence in clinical care, management, and teaching, and no single facet of her professional work can be ranked above the others.

Holding two undergraduate degrees (Pharmacy and Nursing), she devoted herself to psychiatric and mental health care-an area in which she earned a master’s degree and later completed a doctorate and habilitation (*livre-docência*), with training at public institutions in Brazil and abroad. As a nurse, she had the opportunity to organize a psychiatric ward at Hospital das Clínicas in Salvador, a landmark in establishing inpatient care for people experiencing mental distress within a general hospital, where she sought new approaches beyond the asylum system. At the time (the 1950s), Brazil was still far from initiatives aimed at deinstitutionalization in psychiatry; nevertheless, Tereza Sena embraced a transformative perspective. Asylum-based care, profitable for clinic owners, required staff to maintain rigid, disciplinary routines and silenced service users’ voices, so length of stay was not a concern. Asylums were overcrowded and offered poor conditions of care, especially in nursing care^(3,4)^.

For Tereza, the Juqueri Hospital-Colony became a setting that demanded commitment to what seemed impossible. Patients were kept in appalling conditions and subjected to violence, abuse, and mistreatment. There was no rehabilitation; instead, crises worsened. In addition, many of those institutionalized were political prisoners and people excluded by society who, in most cases, did not have mental disorders but became ill due to confinement; these problems led to the hospital’s closure years later^(11)^.

In that setting, without losing her composure and using a cart to move through the pavilions, Tereza organized herself to train and supervise nursing teams responsible for 11,000 institutionalized patients. She called attention to horrific asylum-based practices that she witnessed, criticized, and tried to undo. With characteristic patience, she gradually sought to transform the nursing staff and other workers through knowledge, ongoing dialogue, and her example. Her exemplary presence earned the respect and affection of the people with whom she lived and worked.

She also understood that clinical practice should be closely tied to teaching-that is, the faculty member should work within the institution that served as the students’ clinical placement site, providing a concrete model of what nursing practice should look like^(4)^.

At the time, finding faculty with experience in psychiatry was a major challenge. Since the enactment of Law No. 775 in 1949, which restructured nursing education in Brazil, nursing schools were required to offer clinical placements in different areas, including at least 15 days in neurology and psychiatry^(29)^. This requirement created a problem for most schools because there were very few instructors with the experience needed to supervise students in these settings.

This challenge was compounded by the fact that the schools were all-female and operated under a residential (boarding) system, with an emphasis on preserving moral standards and safeguarding students’ integrity. Under these conditions, upholding those commitments became impractical in psychiatric institutions, which were overcrowded and lacked formally trained nurses. By then, public criticism of asylums was already mounting; critics pointed to their therapeutic ineffectiveness and growing problems in infrastructure and workforce training, which helped fuel the Anti-Asylum Movement. The anti-asylum movement gained strength worldwide from the 1960s onward; in Brazil, however, the influence of the psychiatric reform movement in Trieste, Italy, was more pronounced, although precarious conditions in psychiatric institutions persisted into the 2000s^(3,4)^.

For Tereza Sena, the Anna Nery School of Nursing was her home for most of her working years. There, she became well known as a psychiatric nursing professor in the undergraduate program and as a faculty member and advisor in graduate programs, producing influential studies that advanced the field.

Throughout the 20th century, the Anna Nery School remained one of the country’s leading institutions. Responsible for disseminating modern nursing in Brazil, it was consistently held in high regard by most Brazilian nurses because it trained nurses who went on to work across the country and supported the creation of new schools^(3)^.

Concurrently with her teaching at the Anna Nery School of Nursing, Tereza Sena was appointed director of nursing at IPUB, marking the first time that service included a degree-prepared nurse who also held teaching responsibilities in psychiatric nursing. There, she helped establish a clinical training site for students and faculty from different nursing schools, revealing a spiral of knowledge dissemination. Through a transformative approach to care, she gradually influenced professionals who would later engage in the psychiatric reform movement. IPUB originated as the former observation pavilion of the Pedro II Asylum (Hospício Pedro II) and was intended to provide free evaluations for individuals referred by public authorities and suspected of insanity. In 1938, it was transferred to the newly created UB, becoming that university’s Institute of Psychiatry^(11)^.

At IPUB, Tereza Sena had the opportunity to underscore the nurse’s role in care that upheld the freedom of people experiencing mental distress. The aim was to humanize care in line with an emerging paradigm in developed countries where isolating patients and denying their rights could no longer be regarded as “care.” Her former students were hired to join IPUB’s nursing staff and, together, they introduced broader, more participatory care, with innovative solutions better suited to psychiatric treatment, overcoming resistance from the nursing staff itself^(4)^.

Biographical studies offer a range of information that can take researchers down unexpected paths. The biography of Tereza de Jesus Sena-known as “Tereza Sena,” “Dona Tereza,” or “Tetê”-revealed her legacy as a professor and nurse in psychiatric and mental health care, as well as the paths she forged to work differently from the prevailing institutional model, amid countless challenges. She drew on knowledge and practices that broke with prevailing norms, seeking to ensure greater dignity for people experiencing mental distress. Through her example as a nurse and in her teaching, she sought to transform the collective around her: health teams, as well as students and faculty from other institutions. Her purpose was to show that other forms of care and nursing education were possible.

### Study limitations

Although this study reveals numerous aspects of Tereza de Jesus Sena’s professional work, it offers only a partial account of her achievements, with gaps regarding her political engagement within nursing and her contributions to scholarly output. It should also be acknowledged that the lack of organized nursing archives limited the documentary corpus, prompting the development of new projects that can question or complement this biography.

### Contributions to the field of nursing, health, and public policies

The contributions of a biographical study of Tereza Sena to nursing history span both psychiatric and mental health nursing practice and teaching. The knowledge produced helps clarify part of Brazilian nursing’s trajectory, which, under her leadership, was transformed to dismantle asylum-based practices and improve the quality of teaching.

## FINAL CONSIDERATIONS

For a long time, psychiatric and mental health nursing was not regarded by nurses as a priority area of professional practice. Trajectories such as that of Tereza de Jesus Sena sparked innovations in practice and teaching in the field, drawing nurses to the care of people experiencing mental distress.

Throughout her academic and professional life, she applied her training and became an agent of change. She faced prejudice and pushback and remained open to dialogue with other professionals to show that it was possible to provide better conditions of care within psychiatric institutions and strengthen bonds with patients and families. She established health services, led teams, reorganized care routines and protocols, and reformed nursing education in the field.

This professional taught psychosocial care 30 years before the Brazilian Psychiatric Reform introduced the term. She was a woman and a professor ahead of her time. With intelligence and unwavering ethical commitment, Tereza Sena moved through each stage of her career by elevating nursing care and teaching, opening space for new ideologies and models in psychiatric and mental health nursing.

## Data Availability

The research data are available within the article.
