# Supplementary material for: Tereza de Jesus Sena: reformer of psychiatric and mental health nursing education and practice
Source: Rev Bras Enferm. 2026 Jul 24;79:e20250223. doi: 10.1590/0034-7167-2025-0223 (PMC13403638; doi:10.1590/0034-7167-2025-0223)
Supplement: Translation

# Translation: Tereza de Jesus Sena: reformadora do ensino e prática em enfermagem psiquiátrica e saúde mental

**DOI:** 10.1590/0034-7167-2025-0223pt

**Authors:** Maria Angélica de Almeida Peres, José Lúcio Costa Ramos, Priscilla Ingrid Gomes Miranda, Lygia Paim, Cristina Maria Douat Loyola

**Affiliations:** I Universidade Federal do Rio de Janeiro. Rio de Janeiro, Rio de Janeiro, Brasil; II Universidade Federal da Bahia. Salvador, Bahia, Brasil; III Universidade Ceuma. São Luís, Maranhão, Brasil

**Keywords:** Enfermagem, Biografia, História da Enfermagem, Enfermagem Psiquiátrica, Saúde Mental.

## Abstract

**Objetivos:**

descrever a biografia de Tereza de Jesus Sena, destacando sua contribuição ao ensino e prática em enfermagem psiquiátrica e saúde mental.

**Métodos:**

estudo biográfico, cuja análise histórica triangulou fontes e interpretou contextualizadamente os dados, organizados cronologicamente. A coleta ocorreu de agosto a dezembro de 2023.

**Resultados:**

emergiram três categorias: Dados pessoais, formação e início da carreira profissional; Efetivação e reconhecimento como docente de Enfermagem Psiquiátrica; e Relação de Tereza de Jesus Sena com o desenvolvimento da enfermagem psiquiátrica no Brasil.

**Considerações Finais:**

a enfermeira cearense destacou-se pela atuação nos estados da Bahia, São Paulo e Rio de Janeiro. Foi uma profissional que investiu no trabalho e na difusão do conhecimento pela educação como ferramentas de transformação do campo psiquiátrico e de saúde mental, tornando-se a mentora de um cuidado centrado na subjetividade e de um ensino moderno para sua época.

## INTRODUÇÃO

Atualmente, o campo da saúde mental configura-se como interdisciplinar, com equipes atuando em determinado território com a responsabilidade de prestar assistência voltada ao bem-estar da pessoa em sofrimento psíquico^(1)^. A compreensão dos avanços responsáveis pela consolidação da enfermagem em saúde mental neste século XXI requer estudos que forneçam elementos para contextualizar o desenvolvimento dos saberes e práticas. Tais elementos ajudam a explicar como a enfermagem brasileira assumiu protagonismo nesse cuidado transferido das instituições psiquiátricas para dispositivos na comunidade.

A Luta Antimanicomial no Brasil tem registros desde a década de 1960, quando a sociedade - representada por usuários, familiares e trabalhadores de saúde mental - voltou-se contra uma realidade desumana e incoerente da assistência psiquiátrica. As práticas manicomiais foram denunciadas nos diferentes meios de comunicação e ecoaram como pedido de transformação, ainda em curso^(2)^.

Foi na década de 1960 que uma enfermeira dedicada às pessoas em sofrimento psíquico ganhou visibilidade nacional pela coragem, inteligência e competência, que a colocaram entre as principais profissionais de seu tempo. Tereza de Jesus Sena (em algumas fontes, Teresa) foi um exemplo para gerações que puderam conhecer seu trabalho como enfermeira, professora, pesquisadora e gestora; certamente, a sua biografia será capaz de iluminar e animar gerações futuras de profissionais para o campo do cuidado em saúde mental^(3)^.

A justificativa para a realização deste estudo biográfico encontra-se na necessidade de ressaltar as contribuições de Tereza de Jesus Sena para a enfermagem brasileira, sobretudo para a enfermagem psiquiátrica e de saúde mental. O intuito é registrar sua memória e disponibilizar para a sociedade o conhecimento sobre sua vida dedicada ao cuidado especializado; essa entrega se deu quando as transformações no cenário das práticas em saúde mental ainda estavam cerceadas pela cultura manicomial, que, por meio da instituição psiquiátrica, alijava da sociedade as pessoas em sofrimento psíquico^(2)^.

Seu trabalho ainda serve como base para oferecer dignidade às pessoas internadas em hospitais psiquiátricos e para sensibilizar futuros profissionais pelo ensino de enfermagem psiquiátrica; nesse movimento, expõe uma realidade gradativamente transformada por lutas coletivas e individuais, travadas em prol do reconhecimento da especialidade e da minimização do estigma direcionado aos profissionais dessa área^(4)^. Ela foi uma mulher que obteve reconhecimento pela competência de seu trabalho, elevando a prática assistencial pelo conhecimento especializado, pela pesquisa científica e pela capacidade de compreender o outro, fosse ele a pessoa em sofrimento psíquico, fosse sua família ou mesmo o profissional de saúde.

## OBJETIVOS

Descrever a biografia de Tereza de Jesus Sena, destacando sua contribuição ao ensino e à prática em enfermagem psiquiátrica e saúde mental.

## MÉTODOS

### Aspectos éticos

Por se tratar de pesquisa histórica em fontes documentais de acesso público, a aprovação em Comitê de Ética em Pesquisa foi dispensada.

### Tipo de estudo

Estudo biográfico^(5)^, de abordagem qualitativa. Nesse tipo de estudo, detalham-se características típicas de uma pessoa cuja trajetória colaborou para o conhecimento do coletivo e para o desenvolvimento da ciência^(6)^. Foram utilizados os critérios propostos pelos Padrões para Relatórios de Pesquisa Qualitativa (SRQR)^(7)^.

### Coleta e organização dos dados

O corpus documental foi composto por histórico escolar, relatórios, livros de registros, fotografias e convite de formatura, consultados no Museu da Escola de Enfermagem da Universidade Federal da Bahia. Também foram incluídos discursos, artigos, reportagens, ofícios e duas entrevistas concedidas pela biografada (uma em 1990 e outra em 2002), pertencentes ao acervo do Centro de Documentação da Escola de Enfermagem Anna Nery - Universidade Federal do Rio de Janeiro. A coleta de dados ocorreu entre agosto e dezembro de 2023.

### Análise dos dados

A análise de dados seguiu a triangulação de fontes, realizada após a seleção, organização cronológica e categorização dos documentos. Os dados foram agrupados por temas: dados biográficos, formação educacional e atuação profissional. A etapa final foi a descrição e interpretação histórica, levando à construção do texto em três categorias: Dados pessoais, formação e início da carreira profissional; Efetivação e reconhecimento como docente de Enfermagem Psiquiátrica; e Relação de Tereza de Jesus Sena com o desenvolvimento da enfermagem psiquiátrica no Brasil.

## RESULTADOS

### Dados pessoais, formação e início da carreira profissional

Tereza de Jesus Sena, filha de Francisco Sena Araújo e Júlia Adélia Araújo, nasceu no dia 8 de outubro de 1923, na cidade de Acaraú, no estado do Ceará - localizada a 238 km da capital, Fortaleza. Na capital, concluiu o (então chamado) ensino primário no Colégio Estadual do Ceará e o ensino secundário na Escola Normal Justiniano de Serpa, instituições tradicionais e renomadas do estado^(8)^.

Sua primeira formação de nível superior foi em Farmácia, na Faculdade de Farmácia e Odontologia do Ceará. Tornou-se enfermeira pela Escola de Enfermagem da Bahia, residindo no internato da escola. Em seu histórico escolar, consta que ingressou no curso em 15 de março de 1949 e colou grau em 9 de dezembro de 1952. Nessa época, seus pais já tinham falecido.

Durante suas folgas, segundo registro no Livro de Entrada e Saída de Alunas do prédio do internato (1950-1951), Tereza Sena costumava sair para ir aos correios, ao centro da cidade, ao cinema e ao Farol da Barra; essas idas indicam que se comunicava com familiares e amigos por cartas e que tinha interesse em passeios culturais.

Sua formatura e das demais 25 colegas de turma ocorreu em solenidade de Colação de Grau e cerimônias religiosas nos dias 9 e 10 de dezembro de 1952. Recém-formada, realizou treinamento de um mês na Clínica Psiquiátrica da Universidade de São Paulo (USP). Ao retornar para a Bahia, foi contratada como professora da Escola de Enfermagem da Universidade Federal da Bahia (UFBA), onde ministrou as disciplinas de Enfermagem Psiquiátrica, Neurologia e Enfermagem Neurológica, entre 1953 e 1955. Além disso, acompanhou alunas no estágio da disciplina de Enfermagem Psiquiátrica no Hospital Juliano Moreira, fundado em 1869, na cidade de Salvador^(9)^.

De 1954 a 1955, Tereza Sena foi Chefe do Serviço (Divisão de Enfermagem) do Hospital Universitário Professor Edgard Santos da UFBA, também conhecido localmente como Hospital das Clínicas. Associou-se ao médico catedrático de psiquiatria da Faculdade de Medicina da Bahia, Nelson Soares Pires, reconhecido por iniciar a reforma psiquiátrica baiana, organizando com ele a Unidade Psiquiátrica do Hospital das Clínicas de Salvador. Inaugurada em 1953, essa unidade foi o primeiro serviço de psiquiatria em Hospital Geral do Brasil, constituído por uma enfermaria exclusivamente feminina, que recebia pacientes de todas as classes sociais. Tereza Sena, com seu potencial inovador, tornou-se responsável pelo serviço de enfermagem nessa enfermaria psiquiátrica, e o atendimento médico incluía consulta ambulatorial, realizada no próprio hospital^(10)^.

Em 1956, ela foi contratada pelo Departamento de Assistência ao Psicopata (DAP) de São Paulo para ser chefe de enfermagem do Hospital Central do Juqueri, uma instituição criada no século XIX, que funcionava nessa época como hospital-colônia e comportava 5.210 leitos. O Juqueri foi referência no Brasil durante os anos de 1923 a 1938, sob a direção de Antônio Carlos Pacheco e Silva. Suas instalações foram progressivamente ampliadas e contava com equipe de médicos generalistas, neurologistas e psiquiatras^(11)^. Quando Tereza Sena chegou para atuar como enfermeira, o Juqueri apresentava uma superlotação duas vezes maior que sua capacidade: eram mais de 11 mil internos, distribuídos em 12 pavilhões com alas masculina, feminina e infantil, asilo e centro médico. Ainda oferecia serviços de manicômio judiciário, cemitério, entre outros^(12)^.

Durante sua atuação no Hospital Colônia do Juqueri, conheceu a enfermeira norte-americana Verna Fraser, que foi trazida ao Brasil pelo Serviço Especial de Saúde Pública (SESP) para atuar por dois anos como consultora de enfermagem psiquiátrica nas escolas de enfermagem, uma vez que o estágio em psiquiatria tinha passado a ser obrigatório. A consultora americana indicou Tereza para cursar o mestrado em Enfermagem Psiquiátrica, com bolsa de estudos financiada pelo governo dos Estados Unidos da América. Tereza Sena contou sobre seu encontro e convívio com essa enfermeira:

*Eu estava lá [no Juqueri] tinha pouco tempo, chegou uma consultora de enfermagem psiquiátrica americana: Verna Fraser era o nome dessa consultora; ela não falava uma palavra de português e eu não falava inglês, e nem entendia, eu comecei a aprender com ela [...]. E nós trabalhávamos também até tarde da noite porque ela ia por períodos lá para São Paulo [...]. E um belo dia, ela me perguntou assim, de súbito: Tereza, se eu conseguir uma bolsa do governo americano para você ir para os Estados Unidos, você gostaria de ir fazer um curso? Se você aceitar, será a minha indicada* ^(13)^.

Após dez meses de dedicação ao idioma inglês, Tereza Sena foi entrevistada pelos representantes do governo dos Estados Unidos e, em janeiro de 1958, embarcou para a cidade de Detroit, no estado de Michigan. Foi a única enfermeira da psiquiatria indicada para a bolsa de estudos nesse período. Fez o curso de mestrado em Enfermagem Psiquiátrica no *College of Nursing/Wayne State University*, além de estágios no *St. Elizabeth’s Hospital*, em Washington, e no *Health, Education and Comfort Department*, em Nova Iorque. Regressou ao Brasil em 1959, tornando-se enfermeira-chefe do Hospital do Juqueri e supervisora de estágio das alunas da Escola de Enfermagem da USP, consolidando seu conhecimento e sua carreira na área de enfermagem psiquiátrica e saúde mental.

### Efetivação e reconhecimento como docente de Enfermagem Psiquiátrica

A carreira docente de Tereza Sena iniciou na Bahia logo após sua formatura, simultaneamente à sua experiência assistencial. Quando foi trabalhar em São Paulo, passou a supervisionar estudantes da Escola de Enfermagem da USP em estágio no Hospital Colônia do Juqueri, mantendo a associação entre ensino e prática assistencial.

Entretanto, Tereza tinha o desejo de se mudar para o Rio de Janeiro e ingressar como docente na Escola Anna Nery da Universidade do Brasil (UB), uma referência nacional naquele momento. Isso se concretizou de forma inesperada, com um telefonema da diretora da escola, Waleska Paixão, para o Hospital Colônia do Juqueri, a fim de falar com a chefe de enfermagem. Tereza Sena contou que a conversa foi direta:

*Dona Waleska me chamou e eu fui ao Rio de férias; ela conversou comigo, disse que estava precisando de professora [...]. Mas eu disse que primeiro eu queria passar todo o meu trabalho. Eu já tinha conseguido seis enfermeiras lá [no Juqueri], mas a D. Waleska disse que não podia esperar, então eu deixei uma enfermeira no meu lugar* ^(13)^.

Sua ida para a Escola Anna Nery foi dificultada pela negativa do DAP-SP em liberar sua transferência. Em 31 de dezembro de 1959, após negociações da diretora Waleska Paixão, Tereza foi contratada para integrar a Campanha Nacional Contra a Tuberculose (CNCT), que podia admitir funcionários por um ano, a título precário. O acordo entre a Escola Anna Nery e a CNCT era a cessão de Tereza em troca da enfermeira Ariadne Lopes de Menezes, há tempos cedida pela Escola à Campanha.

Em abril de 1960, Tereza Sena começou a ministrar a disciplina de Enfermagem Psiquiátrica na Escola Anna Nery. Sua inserção não foi fácil, em razão de sua origem nordestina e formação em outra escola, como relatou em sua entrevista, ao lembrar como foi recepcionada pelas professoras da Escola Anna Nery:

*Aqui pra nós, [fui recebida] muito mal, indiferente, socialmente não queriam conversa e até faziam críticas. Às vezes eu sabia através das alunas, mas eu sempre ignorava, nem com a D. Waleska eu comentava [...] houve um boicote velado* ^(14)^.

A despeito da rejeição que recebeu das colegas, seguiu dedicando-se ao ensino de Enfermagem Psiquiátrica, enquanto a diretora da escola empreendia esforços para efetivá-la por meio de negociações com os diretores de serviços de saúde e com o reitor da UB. Em fevereiro de 1961, foi nomeada encarregada de curso do quadro extraordinário da UB. Foi contratada inicialmente pelo período de três meses, recebendo por verba especial. Tal situação foi mantida mediante renovações de contrato, de quatro em quatro meses, até o ano de 1962, quando foi empossada no cargo de enfermeira do quadro extraordinário da UB^(3)^.

Já sua inclusão no quadro ordinário de funcionários ocorreu em 1969, em razão da Lei nº 3.780, de 12 de julho de 1960, atualizada pela Lei nº 4.069, de 11 de junho de 1962, a qual determinou que os servidores com cinco anos de efetivo exercício em atividade de caráter permanente fossem enquadrados nos cargos e funções da universidade^(15)^.

Tereza Sena passou a substituir os professores médicos ministrando as disciplinas Higiene Mental e Fundamentos Clínicos de Psiquiatria (nova denominação dada à antiga disciplina de Psiquiatria). Ao avaliar o ensino das disciplinas até aquele momento, a professora descreveu como “cinquenta anos atrasado”^(14)^.

Atualizou o ensino com novas perspectivas teóricas e práticas sobre o cuidado às pessoas em sofrimento mental institucionalizadas, além de desenvolver uma relação afetiva com as alunas, chegando a inspirá-las para seguir a área de psiquiatria. Uma ex-aluna entrevistada em outra pesquisa referiu:

*Adorei o curso de enfermagem [...]. E, aí, veio... quer dizer, eu já tinha uma tendência, e veio uma pessoa que realmente me ajudou a decidir, que foi Tereza Sena. Ela dava aquelas aulas maravilhosas, e do convívio que eu tive com Tereza, eu já me inclinei. Então, o destino é esse [a atuação como enfermeira psiquiátrica]* ^(13)^.

A professora Tereza Sena também inspirava as futuras enfermeiras pela aparência. Vestia-se com uniforme branco e cabelos alinhados. Ensinava com isso a importância da harmonia da estética pessoal, ainda que lidando com a desorganização da loucura em algumas situações. Ela demonstrava bom humor com os pacientes, por vezes rindo com eles sobre suas histórias. Nunca ria dos pacientes, o que atestava sua ética profissional.

Ela detinha estudos em psicanálise^(4)^, de modo que, nos anos de 1970, ensinava utilizando conceitos de Jacques Lacan. Orientava as alunas sobre o modo de secretariar a loucura dos pacientes esquizofrênicos, alguns de difícil abordagem; e partia do pressuposto de que quem sabia sobre si era o paciente. Quem o acompanhava deveria segui-lo em seu percurso de delírios ou de alucinações, já que seus comportamentos davam um testemunho de sua saúde mental. Todas as manhãs, ao final do estágio, ocorria uma reunião com a professora para discutir casos e condutas realizadas pelas estudantes.

No contato com os pacientes, ela aplicava o Modelo de Relação Pessoa-a-Pessoa, da enfermeira Joyce Travelbee, cuja intuito era auxiliar indivíduos a lidarem com a experiência da doença e do sofrimento e, se possível, encontrar sentido nessas experiências, sendo o objetivo final a presença da esperança. As alunas aprendiam que o cuidado em saúde mental não era épico, ou seja, não havia uma chegada triunfal à cura. Com os pequenos avanços, reconheciam-se as possibilidades de cada paciente.

Na pós-graduação da Escola Anna Nery, inaugurada em 1972 com o curso de mestrado, Tereza Sena compôs o quadro de docentes pioneiras - juntamente com as professoras Elvira de Felice Souza, Maria Dolores Lins de Andrade, Cilei Chaves Rhodus e Vilma de Carvalho - na qualidade de encarregadas pelo planejamento e implantação desse curso. Colaborou com a formação stricto sensu em enfermagem ao orientar pesquisas no campo da psiquiatria e em outras áreas^(16)^. Em 1989, com a criação do doutorado em enfermagem na EEAN, ela iniciou a orientação de teses. Entre 1983 e 1990, ocupou o cargo de coordenadora do Programa de Pós-Graduação Stricto Sensu da EEAN.

Tereza Sena também exerceu importante influência no desenvolvimento da pós-graduação em Enfermagem na Universidade Federal do Ceará (UFC), durante a fase de consolidação da pós graduação stricto sensu no país. Sua participação como orientadora e consultora científica em pesquisas do programa evidenciou o diálogo institucional então estabelecido entre centros formadores do Sudeste - especialmente a EEAN/UFRJ - e iniciativas emergentes no Nordeste. Essa parceria contribuiu para a circulação de referenciais teórico-metodológicos, qualificação dos estudos produzidos e formação de quadros docentes locais.

Além disso, ela participou ativamente de eventos científicos como organizadora, ministrante de cursos, debatedora em grupos de discussão e apresentadora de trabalhos^(17)^; publicou livro sobre enfermagem psiquiátrica^(18)^; publicou artigos^(19,20)^; integrou a comissão avaliadora de trabalhos na Revista Brasileira de Enfermagem^(21)^; e participou de comissões examinadoras de concurso público docente^(22,23)^ e de concurso de habilitação à livre-docência^(24)^. Além disso, fez parte da Comissão de Especialistas de Ensino de Enfermagem do Ministério da Educação e Cultura (MEC) e atuou como consultora do Departamento de Assuntos Universitários^(25)^. Um dos feitos de Tereza Sena nessa posição foi o estudo de viabilização e implantação do curso de Enfermagem da UFC, criado em 1970^(26)^.

A docência e a prática como enfermeira no campo da enfermagem psiquiátrica e de saúde mental sempre caminharam juntas na trajetória de Tereza Sena, na Bahia, em São Paulo e no Rio de Janeiro. Eram ações que se complementavam e geravam impactos positivos nos serviços de saúde e na formação de enfermeiras. Foram muitas homenagens como paraninfa das turmas de graduação, escrevendo discursos para essas ocasiões sobre temas relacionados à enfermagem psiquiátrica, que foram arquivados pela EEAN como fontes primárias e merecem ser objeto de novas pesquisas históricas.

### Relação de Tereza de Jesus Sena com o desenvolvimento da enfermagem psiquiátrica no Brasil

No processo de qualificação do ensino na Escola Anna Nery, a articulação entre a assistência e o ensino vinha sendo promovida pela nomeação de enfermeiras e professoras da Escola na chefia dos hospitais, assim como ocorria no Hospital São Francisco de Assis, na Maternidade-Escola e no Instituto de Neurologia da UB. Sendo assim, após a efetivação de Tereza Sena na universidade, a diretora Waleska Paixão articulou com o diretor do Instituto de Psiquiatria da UB (IPUB), José Leme Lopes, a indicação da referida professora à chefia de enfermagem^(14)^. Cabe destacar que, até aquele momento, o IPUB nunca havia tido em seu quadro uma enfermeira diplomada^(4)^.

A partir de 1962, já como chefe de enfermagem do IPUB, Tereza Sena passou a reformular a assistência de enfermagem psiquiátrica na instituição, em especial nas enfermarias de internação; esse setor ainda se mantinha distante do conceito da Nova Psiquiatria, já vigente em outros países (p.ex., Estados Unidos), onde ela havia feito seu curso de mestrado e de onde havia trazido uma nova concepção de enfermagem psiquiátrica^(27)^.

Ao mesmo tempo, ela iniciou uma reconfiguração do espaço assistencial do IPUB que era contrária ao modelo manicomial praticado ali:

*Para eles [equipe de enfermagem] o que valia era medicar o paciente, amarrar o paciente, trancar no quarto forte, e ter na cabeça que o paciente era uma pessoa paciente* ^(13)^.

Tereza Sena contou como negociou o fim do quarto-forte:

*Um dia cheguei para o administrador e perguntei por que naquela época ainda tinha quarto forte. Ele falou: ‘É que aqui não tem pessoa que se responsabilize.’ Aí eu falei pra ele que assumia [...]. Aí, várias noites eu ia pra lá pro Instituto e ficava à noite de plantão, para os doentes poderem dormir com o quarto aberto* ^(14)^.

Além do fechamento do quarto-forte, ela fez outras modificações na assistência, para aproximar a enfermagem do IPUB ao que já se fazia em países mais avançados. Para tanto, enfrentou um movimento de resistência da equipe de enfermagem e de nutrição:

*Tudo era proibido, [os funcionários da cozinha] riam e achavam tudo coisa de doido, ninguém comia no prato, nem com colher [...]. Eu comecei também a mostrar para a nutricionista que eles podiam comer com talher. [...] elas foram experimentando e eu em cima* ^(14)^.

As novas práticas de cuidado envolviam considerar as pessoas internadas em sua individualidade, sem que a vigilância fosse coercitiva, incluindo passeios e outras atividades como oficina de horta. Tereza Sena recebia as alunas na parte da manhã e cuidava, à tarde, da administração do serviço de enfermagem^(14)^. Também teve influência na contratação de enfermeiras formadas pela Escola Anna Nery para o IPUB, construindo uma identidade profissional dentro de uma moderna visão de mundo no tocante ao cuidado de enfermagem às pessoas com transtornos psíquicos.

Suas ações repercutiram nas escolas de enfermagem do Brasil, que passaram a pedir filiação à Escola Anna Nery para que suas alunas pudessem ter acesso às disciplinas de Enfermagem Psiquiátrica e Arte da Enfermagem^(4)^. Nesse cenário, as escolas que se filiaram foram: Escola de Enfermeiras Luiza de Marillac (Pontifícia Universidade Católica do Rio de Janeiro), Escola de enfermagem Alfredo Pinto (Ministério da Saúde) e Escola de Enfermagem Wenceslau Brás (Itajubá, Minas Gerais).

Em 1963, devido ao elevado número de filiações, foi acordado que as diretoras das escolas enviariam primeiro as professoras para um treinamento e depois as alunas. Dessa forma, as próprias professoras supervisionavam o estágio no IPUB, mas as aulas teóricas continuavam sendo ministradas por Tereza Sena^(3)^.

A presença das alunas da Escola Anna Nery foi importante para a implantação do novo modelo de enfermagem no IPUB, pois elas, orientadas pela professora Tereza Sena, participaram de um processo de mudança. Com um conhecimento construído em bases psicanalíticas, ela tinha aptidão para facilitar as relações aluna/professora e aluna/paciente, como se observa no trecho de sua fala:

*Elas [as alunas] me amavam. Não sei nem se elas gostavam tanto da enfermagem psiquiátrica, porque elas ainda tinham toda aquela questão de medo do paciente. [...] e todo dia seguinte eu entrevistava pra elas me dizerem o que é que estavam achando, como é que a aluna conseguia transmitir algo para o paciente* ^(14)^.

Percebe-se a atividade educativa incorporada como resultado da subjetividade, cuja percepção era estimulada no diálogo entre professora e estudante. O objetivo era de que a novata compreendesse sua atuação no cenário e sua prática como estagiária, o que funcionava como um acolhimento enquanto demonstrava preocupação da professora com o bem-estar de todas. A consideração do campo subjetivo no ensino e na prática assistencial era uma meta do IPUB naquele momento, quando o corpo médico tentava aplicar mais intensamente os conceitos psicanalíticos, já muito utilizados pelos psiquiatras norte-americanos, introduzindo a pesquisa nessa área e modificando a prática assistencial realizada na instituição^(27)^.

Tereza Sena permaneceu como chefe de enfermagem do IPUB e como docente responsável pelo ensino de enfermagem psiquiátrica durante 18 anos. Sua saída do cargo ocorreu pela necessidade de compor o corpo docente do primeiro curso de mestrado em Enfermagem no Brasil, em 1972, na Escola Anna Nery - nessa época, já denominada Escola de Enfermagem Anna Nery (EEAN).

No cenário associativo, Tereza foi sócia efetiva da Associação Brasileira de Enfermagem (ABEn) por toda a sua carreira, participando ativamente das lutas profissionais. Em 1977, por exemplo, coordenou a Comissão de Temas do XXIX Congresso Brasileiro de Enfermagem em Santa Catarina. Participou também do Conselho Federal de Enfermagem (Cofen), no cargo de conselheira, na diretoria eleita para a gestão de 1985 a 1988, que tinha como presidente a enfermeira Ivanete Alves do Nascimento^(28)^.

No ano 2000, quando voltou a residir em Fortaleza, ela foi homenageada pelo IPUB, que deu seu nome a uma de suas enfermarias, eternizando a memória dessa personalidade e suas contribuições à enfermagem psiquiátrica e de saúde mental.

Tereza de Jesus Sena faleceu com doença de Alzheimer em 24 de maio de 2004, aos 81 anos de idade, no Hospital de Caridade na cidade de Florianópolis, Santa Catarina. Seu corpo foi transladado para Fortaleza, Ceará, onde foi sepultada.

Em 2023, a Academia Cearense de enfermagem (ACEN), criada em 12/02/2011, resgatou a história de enfermeiras que foram importantes para a assistência, ensino e pesquisa no estado, por meio da publicação do livro Academia Cearense de Enfermagem (2007-2023). No capítulo sobre Tereza de Jesus Sena, é retratado que, em 22/08/2013, foi criada a cadeira de número 12, da qual ela é patrona. Atualmente, a ocupante é a professora aposentada Maria de Nazaré de Oliveira Fraga, da UFC, também especialista na área de saúde mental e que foi sua orientanda de mestrado na Escola de Enfermagem Anna Nery entre 1977 e 1981^(28)^.

## DISCUSSÃO

Como personalidade da enfermagem brasileira, Tereza Sena destacou-se nacionalmente em sua vida profissional atuando nos estados da Bahia, São Paulo e Rio de Janeiro, onde contribuiu para o desenvolvimento das práticas e saberes de enfermagem psiquiátrica e de saúde mental. A importância de sua passagem pela enfermagem mostra que ela atuou com igual competência nos âmbitos assistencial, gerencial e docente, não sendo possível hierarquizar essas frentes de trabalho.

Com duas graduações (Farmácia e Enfermagem), ela dedicou-se ao cuidado em psiquiatria e saúde mental, área em que se tornou mestre e doutora livre-docente, com formações em instituições públicas nacionais e internacionais. Como enfermeira teve a oportunidade de organizar uma enfermaria psiquiátrica no Hospital das Clínicas de Salvador, marco inaugural da internação de pessoas em sofrimento psíquico em hospital geral, em que buscou novas formas de cuidado fora da instituição manicomial. Nessa época (década de 1950), o Brasil ainda estava distante de realizações voltadas para a desinstitucionalização no campo psiquiátrico; apesar disso, Tereza Sena se permitia trabalhar com uma perspectiva transformadora. O cuidado manicomial, lucrativo para os donos de clínicas, exigia da equipe a manutenção de uma rotina disciplinadora e ignorava a voz dos usuários, de modo que o tempo de internação não era uma preocupação. Os manicômios eram locais superlotados, com condições precárias de tratamento, especialmente dos cuidados de enfermagem^(3,4)^.

O Hospital Colônia do Juqueri entrou na vida de Tereza como cenário de comprometimento com o impossível. As pessoas internadas eram mantidas em péssimas condições, sofrendo violências e maus-tratos. Não havia reabilitação, e sim piora das crises. Além disso, muitos dos internos eram presos políticos e pessoas excluídas pela sociedade, que em sua maioria não apresentavam transtornos mentais, mas acabavam adoecidas pelo aprisionamento; esses problemas levaram ao fechamento do hospital anos depois^(11)^.

Nesse cenário, sem perder a serenidade e usando um carrinho para se deslocar pelos pavilhões, Tereza se organizava para capacitar e supervisionar equipes de enfermagem responsáveis por 11 mil internos. Ela destacou terríveis práticas manicomiais que viu, criticou e tentou desfazer. Com sua personalidade dotada de paciência, aos poucos buscava transformar a equipe de enfermagem e demais trabalhadores pelo conhecimento, pelo diálogo permanente e pelo exemplo. Sua figura exemplar conquistava o respeito e o afeto das pessoas com quem convivia.

Ela também entendia que a prática deveria estar intimamente relacionada ao ensino, ou seja, a professora deveria estar inserida na instituição que serve de campo prático para as alunas, dando uma verdadeira demonstração de como deveria ser a prática em enfermagem^(4)^.

Encontrar professoras com aderência ao campo da psiquiatria era uma grande dificuldade naquele momento. Desde a promulgação da Lei n° 775, em 1949, que reformulou o ensino de enfermagem no Brasil, tornou-se obrigatório para as escolas de enfermagem oferecer estágio em diferentes áreas, incluindo neurologia e psiquiatria por 15 dias pelo menos^(29)^. Isso fez emergir um problema na maioria das escolas, pois quase não havia professoras com experiência para o acompanhar as alunas nesses cenários.

Somam-se a esse desafio as características das escolas, que eram exclusivamente femininas, com regime de internato e conservação dos padrões morais e da integridade das suas alunas; sendo assim, a manutenção desse compromisso tornava-se inconveniente nas instituições psiquiátricas, por serem superlotadas e não contarem com enfermeiras diplomadas. Nessa altura, a sociedade já desferia críticas ferrenhas ao manicômio, constatando sua ineficácia terapêutica e crescentes problemas de infraestrutura e qualificação de pessoal, abrindo espaço para a Luta Antimanicomial. O movimento antimanicomial ganhou força no mundo a partir da década de 1960; já o Brasil recebeu mais fortemente a influência da reforma psiquiátrica de Trieste na Itália, embora as condições precárias das instituições psiquiátricas permanecessem até os anos 2000^(3,4)^.

Para Tereza Sena, a Escola de Enfermagem Anna Nery foi sua casa durante a maioria dos seus anos de trabalho. Ali ela ganhou notoriedade como professora de enfermagem psiquiátrica no curso de graduação e como docente e orientadora dos cursos de pós-graduação, produzindo relevantes estudos para o desenvolvimento da área.

Ao longo do século XX, a Escola Anna Nery manteve-se como uma das instituições de liderança no país. Responsável por difundir a enfermagem moderna no Brasil, foi sempre uma escola benquista pela maioria das enfermeiras brasileiras, pois formava pessoal para todo o país e apoiava a criação de novas escolas^(3)^.

Concomitantemente à docência na Escola Anna Nery, Tereza Sena foi nomeada para a chefia de enfermagem do IPUB, marcando a primeira inserção, nesse serviço, de uma enfermeira com formação superior e atuação docente em enfermagem psiquiátrica. Ali preparou um campo de prática para estudantes de diferentes escolas de enfermagem, bem como para professoras, revelando um movimento espiral de difusão do conhecimento. Por meio de um cuidado transformador, aos poucos influenciou profissionais que, posteriormente, engajaram-se na reforma psiquiátrica. O IPUB surgiu como o antigo pavilhão de observação do Hospício Pedro II e tinha por finalidade avaliar gratuitamente os suspeitos de alienação enviados pelas autoridades públicas. Em 1938, foi transferido para a recém-criada UB, tornando-se o Instituto de Psiquiatria dessa universidade^(11)^.

Foi no IPUB que Tereza Sena teve a oportunidade de destacar a presença da enfermeira nos cuidados voltados para a liberdade dos sujeitos em sofrimento psíquico. O objetivo era humanizar a assistência sob o ponto de vista de um paradigma assistencial que nascia em países desenvolvidos, onde não era mais possível considerar como cuidado o isolamento e a negação de direitos às pessoas. Suas ex-alunas foram contratadas para a equipe de enfermagem do IPUB e, juntas, introduziram uma assistência mais ampla e participativa com soluções inovadoras e mais adequadas ao tratamento psiquiátrico, superando a resistência da própria equipe^(4)^.

Os estudos biográficos trazem uma gama de informações que leva o pesquisador por caminhos inesperados. A biografia de Tereza de Jesus Sena, “Tereza Sena”, “Dona Tereza” ou “Tetê”, como era conhecida, revelou seu legado como professora e enfermeira no campo da atenção psiquiátrica e da saúde mental, além dos caminhos que criou para atuar de modo distinto do instituído, em meio a inúmeros enfrentamentos. Ancorou-se em conhecimentos e práticas que rompiam padrões, em busca de garantir mais dignidade às pessoas em sofrimento psíquico. Pelo exemplo como enfermeira e no exercício da docência, buscava transformar o coletivo ao seu redor, fossem as equipes de saúde, fossem as alunas e docentes de outras instituições. Tinha o propósito de mostrar que outro cuidado e outra formação em enfermagem eram possíveis.

### Limitações do estudo

Mesmo revelando inúmeros aspectos da atuação profissional de Tereza de Jesus Sena, este estudo alcançou parcialmente os feitos da biografada, com lacunas sobre sua participação política dentro da enfermagem e contribuição para a produção científica. Cabe aqui reconhecer que a ausência de organização de acervos de enfermagem foi uma limitação para um maior corpus documental, instigando o desenvolvimento de novos projetos que possam questionar ou complementar esta biografia.

### Contribuições para as áreas da enfermagem, saúde ou políticas públicas

As contribuições da produção de um estudo sobre a biografia de Tereza Sena para a história da enfermagem perpassam a prática assistencial e docente da enfermagem psiquiátrica e de saúde mental. O conhecimento produzido permite compreender parte do percurso da enfermagem brasileira, que, sob sua liderança, se transformou com vistas à desconstrução de práticas manicomiais e à qualificação do ensino.

## CONSIDERAÇÕES FINAIS

A enfermagem psiquiátrica e de saúde mental foi por um longo período desconsiderada pelas enfermeiras como campo prioritário de atuação profissional. Trajetórias como a de Tereza de Jesus Sena provocaram inovações nos campos da prática e do ensino na área, atraindo enfermeiras para o cuidado às pessoas em sofrimento psíquico.

Em sua vida acadêmico-profissional, ela aplicou seu aprendizado e fez de si mesma um instrumento de mudanças. Enfrentou preconceitos e embates e manteve-se aberta ao diálogo com outros profissionais, para mostrar que era possível fornecer melhores condições de cuidado no contexto institucional psiquiátrico e fortalecer vínculos com pacientes e familiares. Implantou serviços de saúde, liderou equipes, reorganizou rotinas e protocolos de assistência e reformulou a formação em enfermagem na área.

A professora ensinou atenção psicossocial 30 anos antes da cunhagem do termo pela Reforma Psiquiátrica Brasileira. Era uma mulher e professora além do seu tempo. Com inteligência e comprometimento ético inabalável, Tereza Sena cumpriu as etapas de sua carreira mediante o engrandecimento do cuidado e do ensino em enfermagem, abrindo espaço para novas ideologias e modelos na enfermagem psiquiátrica e de saúde mental.

## Data Availability

Os dados de pesquisa estão disponíveis no corpo do artigo.
